# Na-Ag Ion-Exchanged Glass Substrates for Plasmon-Enhanced Fluorescence Imaging of Neutrophils

**DOI:** 10.3390/s25072278

**Published:** 2025-04-03

**Authors:** Vladimir A. Inozemtsev, Maxim E. Dokukin, Yevgeniy M. Sgibnev, Ekaterina A. Sherstyukova, Snezhanna S. Kandrashina, Mikhail A. Shvedov, Artem V. Shelaev, Nikolay V. Nikonorov, Viktoria A. Sergunova, Alexander V. Baryshev

**Affiliations:** 1 Federal Research and Clinical Center of Intensive Care Medicine and Rehabilitology, V.A. Negovsky Research Institute of General Reanimatology, 25 Petrovka St. Bldg. 2, 107031 Moscow, Russia; va.inozemcev@physics.msu.ru (V.A.I.); kmanchenko@yandex.ru (E.A.S.); snezhanna.lyapunova@yandex.ru (S.S.K.); shvedovmike@bk.ru (M.A.S.); 2 Dukhov Automatics Research Institute (VNIIA), 22 Sushchevskaya St., 127055 Moscow, Russia; sgibnevem@yandex.ru (Y.M.S.); artem.shelaev@yandex.ru (A.V.S.); baryshev@vniia.ru (A.V.B.); 3 Research Center for Optical Materials, ITMO University, 49 Kronverksky Avenue, 197101 Saint-Petersburg, Russia; nikonorov@oi.ifmo.ru

**Keywords:** ion exchange, plasmonic nanostructures, silver nanoparticles, neutrophil, fluorescence enhancement and lifetime

## Abstract

Here, we study the fluorescence response of neutrophils stained with the wheat germ agglutinin Alexa Fluor 594 dye when the cells are placed on plasmonic nanoparticle substrates. Specifically, we focused on gold and silver nanoparticles with particle sizes ranging from 12 to 250 nm. It was demonstrated that the intensity of fluorescence can be increased by more than 10 times when using substrates with silver nanoparticles formed by Na^+^-Ag^+^ ion exchange in glass. The fluorescence enhancement depends significantly on both the size and surface density of the silver nanoparticles and the membrane staining procedure.

## 1. Introduction

A growing number of studies have focused on the interaction of plasmonic structures not only with single organic molecules but also with living cells [1,2,3]. Of particular interest are systems that facilitate the generation of surface plasmons, such as rough metallic surfaces or nanoparticles located on a two-dimensional interface. Since the commercialization of surface plasmon resonance microscopy (SPRM) over 25 years ago [4], the technique has become essential in the fields of biomedicine, biosensing, and drug development [5,6]. SPRM is extensively utilized for analyzing the adsorption of molecules on plasmonic surfaces [7], including the development of sensors capable of detecting specific molecules within a particular system [8]. Beyond the detection of individual molecules, SPRM enables the analysis of cellular structures and the investigation of interactions between cells and their environment. For instance, SPRM can be used to study the morphology and cellular adhesion of various cell types [9,10]. Furthermore, SPRM provides the capability to assess cell–substrate interactions at the single-cell level and to monitor cellular responses to external stimuli in real time [11]. Modifications to the SPRM protocol have been proven as an effective label-free analytical approach, allowing for the observation of intracellular dynamics [12,13] and proposed for clinical cellular diagnostics, including the detection of cancer cells [14,15].

In addition to utilizing plasmonic structures as sensors, the plasmonic effect is widely employed to enhance fluorescence intensity. For instance, the brightness of the pyrene excimer was enhanced by a factor of 2.5 in the presence of silver nanoparticles [16]. A similar effect was observed in the case of Rose Bengal dye, where the fluorescence was enhanced by a factor of 2.2 when the dye was combined with gold nanoparticles [17]. The employment of gold and silver nanoparticles, with a diameter ranging from 60 to 100 nanometers, was demonstrated to result in up to fourfold enhancement in coumarin fluorescence [18]. Furthermore, plasmonic enhancement was proposed to increase the sensitivity of single protein detection, achieving a 30-fold improvement in the signal-to-noise ratio [19]. It is important to note that the phenomenon of fluorescence enhancement is conventionally associated with nanoparticles in close proximity to the dye, within a range of 1/20 to 1/2 of the wavelength. Nevertheless, the problem of fluorescence enhancement near a plasmonic surface or at an interface containing plasmonic particles is also of great interest [20,21]. Such systems, which support localized surface plasmon resonances, have been effectively applied to the study of intracellular processes. Specifically, surface plasmon resonance was employed to analyze actin reorganization in cancer cells during adhesion [22]. Furthermore, the dependence of fluorescence intensity in proximity to a silver film on the degree of surface roughness was demonstrated in [23].

The conventional approach for fabricating plasmonic substrates for fluorescence enhancement generally includes the deposition or sputtering of metals, as well as the use of colloidal nanoparticles [21]. A recent study proposed a novel approach involving the Na^+^-Ag^+^ ion exchange in glass, which enables the formation of silver nanoparticle arrays on glass surfaces. This method involves replacing one type of cation, typically Na^+^, in the glass with other cations from a salt melt [24,25]. This allows, for example, the fabrication of surface-enhanced Raman scattering (SERS) sensors by forming silver nanoparticles in the near-surface layer of glass [26], or the formation of silver nanoparticles directly on the surface of Na^+^-Ag^+^ ion-exchanged glass by annealing in a hydrogen atmosphere [27,28]. Moreover, it was demonstrated that silver nanoparticles can be formed on the surface of silicate glass without reducers even under thermal treatment in an air atmosphere [29]. The parameters of the thermal treatment were found to effectively control the size and surface density of the resulting silver nanoparticles. These Na^+^-Ag^+^ ion-exchanged glass substrates have the potential to address the limitations often associated with the short lifetime and reusability of metal plasmonic nanoparticles.

Neutrophils are a biologically relevant model system for substrate studies because these cells can respond rapidly to minimal stimuli, including surface type [30,31,32]. Neutrophils are immune cells that play a crucial role in the body’s primary response to inflammation [33,34]. The investigation of neutrophil cellular processes, including activation [35,36,37], migration, and interaction with other cells [38], is essential for understanding the pathogenesis of numerous diseases [39].

The objective of this study was to investigate changes in the fluorescent response of neutrophil membranes stained with Alexa Fluor 594 conjugated to wheat germ agglutinin when the cells were placed on different types of plasmonic substrates. We examined substrates containing gold nanoparticles obtained through the annealing of a thin gold film (dewetting process) [40], as well as substrates with silver nanoparticles formed via Na^+^-Ag^+^ ion exchange in glass, followed by thermal processing in air.

## 2. Materials and Methods

### 2.1. Substrate Fabrication

Multicomponent silicate glass with the composition 14Na_2_O-5ZnO-3Al_2_O_3_-71.5SiO_2_-6.5F (mol. %) was utilized as the substrate material. The glass synthesis was performed in a quartz crucible at 1450 °C with melt homogenization. The resulting glass slides, with a thickness of 1 mm, were then grinded and polished prior to further use. The Na^+^-Ag^+^ ion exchange process was performed by immersing the glass samples in a salt melt composed of 5% AgNO_3_ and 95% NaNO_3_ (mol. %) at 320 °C for 15 min. Under these ion exchange conditions, the diffusion depth of silver ions was found to be approximately 12 μm [41].

It is important to note that the formation and growth of silver nanoparticles on the glass surface is a multi-step process involving the reduction of silver ions, the diffusion of silver atoms to the surface, and the actual nucleation and growth of nanoparticles. It was observed that the cyclic heating and cooling of the samples result in differences in the density of silver nanoparticles on the surface. Specifically, substrates with a high density of nanoparticles (HDAgNP) were fabricated through four annealing cycles in air at 500 °C for 15 min each, whereas substrates with a lower nanoparticle density (LDAgNP) were prepared by continuously annealing for one hour under the same conditions.

Two types of samples were prepared using the physical vapor deposition process: a control silver film and a thin gold layer. The borosilicate glass (Minimed, Bryansk, Russia) and fused quartz (Siegert Wafer, Aachen, Germany), each measuring 12 × 12 × 1 mm, were used as the substrates. Before deposition, all substrates were cleaned in a Piranha solution (a 3:1 mixture of H_2_SO_4_ and H_2_O_2_) for 15 min. The substrates were then washed with deionized water and dried with dry nitrogen.

The metal deposition was carried out using the Ferri Vatt VATT-700 electron beam evaporation system (Ferri Vatt, Kazan, Russia). Prior to deposition the substrates underwent an additional cleaning in argon plasma at the chamber pressure of 1 × 10^−2^ torr for 20 min. Silver and gold films were deposited at an initial residual pressure of 2 × 10^−5^ torr. To fabricate the gold plasmonic nanoparticles the 6 nm thick gold films (AuNP substrate) were annealed at 500 °C for 90 min in air using the R50/250/12 tube furnace (Nabertherm, Lilienthal, Germany). The deposited silver films, with a thickness of 25 nm (AgFilm substrate), were used without further processing.

### 2.2. Substrate Characterization

The transmission spectra of the samples were measured using a Shimadzu UV-3600 dual-beam spectrophotometer (Shimadzu, Tokyo, Japan) over a spectral range of 250–850 nm with a resolution of 1 nm.

The surface topography of all samples was collected using an NTEGRA Spectra atomic force microscope (NT-MDT, Moscow, Russia). The measurements were performed in the semi-contact mode with a scanning rate of 0.5 Hz and resolution of 20 nm/pixel. The NSG30_SS probes (Tipsnano, Moscow, Russia) with a tip radius of less than 5 nm, a resonance frequency of 320 kHz, and a spring constant of 40 N/m were utilized.

The fluorescence decay time of the Alexa Fluor 594-conjugated wheat germ agglutinin (WGA) dye (catalog no. W11262, Thermo Fisher Scientific, Waltham, MA, USA) was measured using the time-correlated single photon counting method with an SPC-130 module (Becker & Hickl, Berlin, Germany). The WGA Alexa Fluor 594 dye was prepared at a concentration of 1:100 (*v*/*v*) in phosphate-buffered saline (PBS) and added to the surfaces of LDAgNP and HDAgNP plasmonic substrates. A coverslip was placed over the dye solution. The measurements were performed using an NTEGRA Spectra confocal microscope (NT-MDT, Moscow, Russia) equipped with a 473 nm diode laser (pulse duration: 40 ps; pulse repetition rate: 20 MHz) as the excitation source and a PMC100 photomultiplier tube (Becker & Hickl, Berlin, Germany) as the detector. The excitation light was focused onto the sample surface using a 10× M Plan Apo objective (NA = 0.28) (Mitutoyo, Kawasaki, Japan).

### 2.3. Neutrophil Isolation

Neutrophil isolation was conducted under conditions of aseptic sterility. A volume of 6 mL of whole blood was overlaid onto a double-gradient Ficoll solution, containing 3 mL of 1.119 g/cm^3^ and 3 mL of 1.077 g/cm^3^ (Sigma-Aldrich, St. Louis, MO, USA). Centrifugation at 400× *g* for 40 min at room temperature was then performed on a high-speed centrifuge Eppendorf 5804R (Eppendorf, Wesseling-Berzdorf, Germany) without braking. The lower white ring, containing the neutrophils and a small portion of the erythrocytes, was subsequently collected. The erythrocytes were then lysed in ice-cold water for 30 s, followed by the restoration of tonicity using a 2× phosphate-buffered saline (PBS) solution. The lysis procedure was repeated until a complete removal of the erythrocytes was achieved.

The isolated neutrophils were then washed with Hanks’ Balanced Salt Solution (HBSS) and seeded into a culture medium composed of HBSS supplemented with 10 mM HEPES (4-(2-hydroxyethyl)-1-piperazineethanesulfonic acid) and 1% heat-inactivated fetal calf serum (hiFCS). The microscopic evaluation of the isolated cells confirmed that over 97% were neutrophils, with cell viability exceeding 98%, as verified by trypan blue.

### 2.4. Neutrophil Staining Procedure

In order to stain directly on substrates, the isolated neutrophils were seeded at a concentration of 0.5 × 10^6^ cells/mL into 100 mm Petri dishes with plasmonic substrates affixed to the bottom. The cells were then incubated for one hour in an incubator at 37 °C with 5% CO_2_ to allow cell stabilization. The plasma membranes of the neutrophils were labeled with WGA conjugated with Alexa Fluor 594 at a ratio of 1:5000. The dye was added directly to the culture medium and maintained at room temperature for five minutes. Subsequently, the medium was replaced twice with dye-free medium to prevent further cellular staining.

Alternatively, for staining prior to seeding on plasmonic substrates, the neutrophils were stained in suspension immediately after isolation. The plasma membranes were labeled with WGA-Alexa Fluor 594 at a 1:5000 ratio. The dye was added to a cell suspension and kept at room temperature for five minutes. This was followed by two washes with PBS by centrifugation at 400× *g* for 5 min with the subsequent removal of the supernatant. The stained cell suspension was subsequently introduced into the culture medium and seeded at a concentration of 0.5 × 10^6^ cells/mL into 100 mm Petri dishes with plasmonic substrates. The dishes were then incubated for one hour at 37 °C with 5% CO_2_.

### 2.5. Fluorescence Microscopy

A series of fluorescent images of stained cells on different substrate types were captured using a Zeiss Axio Imager M1 wide-field fluorescence microscope (Carl Zeiss, Jena, Germany) equipped with an immersion objective ACHROPLAN 25×/0.8. All measurements were performed under identical exposure parameters and excitation light intensities. To measure the fluorescent signal of neutrophil plasma membranes, the excitation wavelength range was set to 530–585 nm, and images were captured for wavelengths beyond 615 nm. The AxioVision SE64 4.9.1.0 software (Carl Zeiss, Jena, Germany) was utilized for image acquisition. The excitation light intensity was set to 50% of the maximum, and the detector exposure time was 40 ms. Image processing, including intensity quantification, was performed using ImageJ 1.54f software [42]. The fluorescence intensity was analyzed for individual cells at the interface between the cell and the substrate (n = 50).

## 3. Results and Discussion

**Figure 1:**
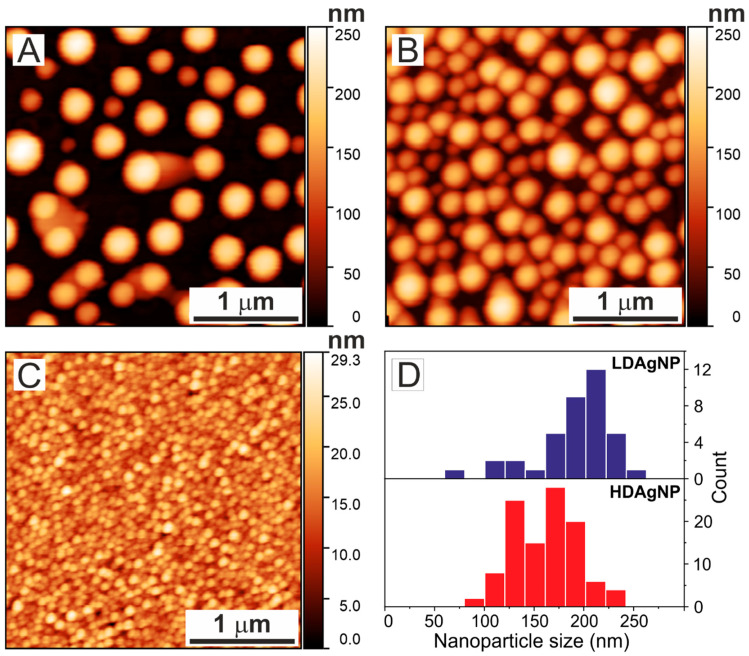
AFM images of the sample surfaces. Panels (**A**,**B**) show LDAgNP and HDAgNP samples, respectively, while panel (**C**) displays the AuNP sample. Panel (**D**) illustrates the height distributions of silver nanoparticles for LDAgNP (upper histogram) and HDAgNP (lower histogram) samples.

Figure 1 shows atomic force microscopy (AFM) images of the surfaces of LDAgNP, HDAgNP, and AuNP samples acquired in semi-contact mode. One can see that the silver nanoparticles (samples LDAgNP and HDAgNP), fabricated using the Na^+^-Ag^+^ ion exchange method, exhibit not only a broad size distribution but the formation of relatively large particles with diameters over 200 nm (Figure 1A,B) [29].

Specifically, the silver nanoparticles formed on the LDAgNP substrate have heights ranging from 76 to 250 nm, with the most frequently occurring value of 201 nm (±28 nm). For the HDAgNP sample, particle heights vary from 92 to 240 nm, with a median value of 165 nm (±27 nm). The HDAgNP sample exhibited a bimodal particle size distribution, with maximums at 130 nm and 170 nm (see Figure 1D).

In addition to substrates with silver nanoparticles, two control samples were examined: a thin silver film deposited on a glass substrate (AgFilm) and a densely packed gold nanoparticle film (AuNP). The 25 nm thick silver film, with a roughness of 1.8 nm, serves as a control to understand the influence of light reflection from the substrate on fluorescence intensity. In the direct geometry employed in this study, incident light can reflect off the nanoparticle-coated substrate, pass through the cells again, and thereby enhance fluorescence intensity.

The substrate with gold nanoparticles (AuNP) used in this study has a mean particle diameter of 12 ± 3 nm and a total thickness of up to 30 nm. These particles exhibit a pronounced plasmon resonance that spectrally aligns with the excitation band of the WGA Alexa Fluor 594 dye.

**Figure 2:**
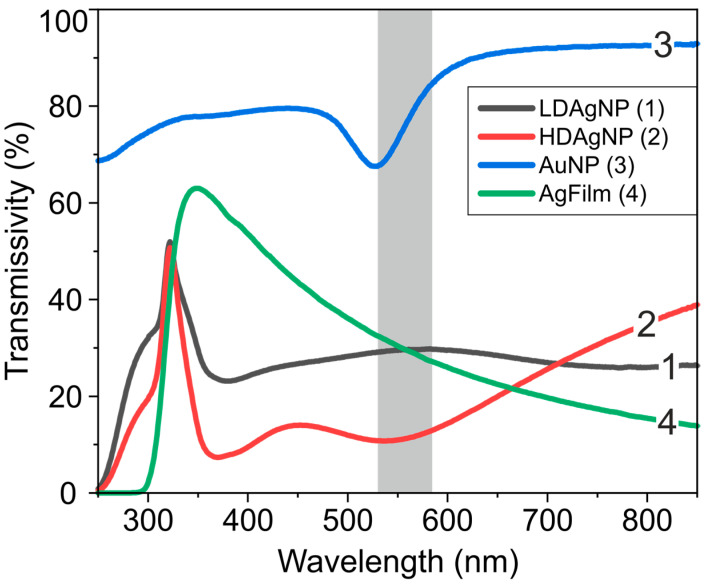
Transmission spectra of LDAgNP (1), HDAgNP (2), AuNP (3), and AgFilm (4) samples. The gray area indicates the wavelength range used for excitation of the WGA Alexa Fluor 594 dye.

The transmission spectra of the LDAgNP, HDAgNP, AuNP, and AgFilm samples are presented in Figure 2. One can see that the transmission spectrum of the thin silver film (AgFilm) exhibits a transmission maximum at 350 nm. This behavior can be associated with two factors: the decrease in the imaginary component of silver’s dielectric permittivity with decreasing wavelength within the 400–1000 nm range [43] and the absorption by the glass substrate. Additionally, a weak peak at 380 nm is observable. This peak is likely connected to interband transitions at the L point of the Brillouin zone [44] or the excitation of surface plasmon-polaritons [43]. The transmission spectrum of the gold film contains spectral features with local minima at 530 nm, 375 nm, and 250 nm. The first minimum corresponds to the localized plasmon resonance of the gold nanoparticles [45]. The shorter wavelength and lower amplitude bands are related to the spectral characteristics of the imaginary part of gold’s dielectric permittivity and correspond to interband transitions at the X and L points of the Brillouin zone, respectively [46].

The transmission spectra of the LDAgNP and HDAgNP samples are characterized by broad, significantly overlapping absorption bands in the visible region, with minima around 310 nm, 380 nm, and 790 nm for LDAgNP, and 305 nm, 370 nm, and 540 nm for HDAgNP. The short-wavelength band is associated with intraband transitions in thin silver films [43,47] and overlaps with the absorption band of residual Ag^+^ ions in the Na^+^-Ag^+^ ion-exchanged glass substrate with the peak at 225 nm [48,49]. The longer wavelength bands in the transmission spectra of LDAgNP and HDAgNP are associated with the localized plasmon resonance of silver nanoparticles. It is well known that the extinction spectrum of nanoparticles significantly depends on their size and shape. For nanoparticles much smaller than the wavelength, typically with diameters below 50 nm, the electromagnetic field is almost uniformly distributed across the nanoparticle’s cross-section. As a result, all conduction electrons oscillate in phase, leading to a single absorption band corresponding to dipolar oscillations [50]. However, as the nanoparticle size increases, the electromagnetic field of the incident wave becomes unevenly distributed, causing phase delays in the electron cloud oscillations. This, in turn, leads to the broadening of the dipolar peak and the excitation of multipolar resonances (quadrupolar, octupolar, etc.), depending on the size and spatial arrangement of the particles [51,52]. Consequently, the absorption band corresponding to dipolar oscillations shifts toward the longer wavelength region. Given that the LDAgNP and HDAgNP surfaces contain particles with sizes comparable to the wavelengths in the visible range (Figure 1A,B), their extinction spectra include bands corresponding to higher-order multipolar oscillations. It is important to note that these bands are significantly broadened due to the size heterogeneity of the nanoparticles. Furthermore, the interaction between nanoparticles that are in close proximity can influence the transmission spectrum [52,53]. Therefore, the primary contribution to the enhanced fluorescence response from the cells is expected to originate from plasmon resonances occurring within the spectral range corresponding to dye absorption (530–585 nm in this study).

**Figure 3:**
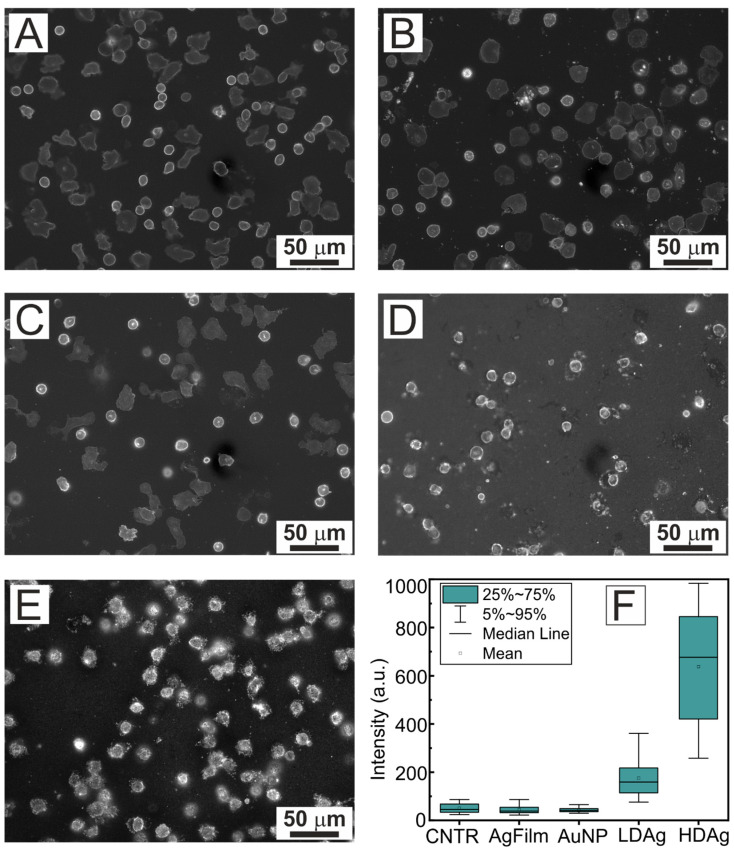
Fluorescence images of neutrophils seeded on different substrates: CNTR (**A**), AgFilm (**B**), AuNP (**C**), LDAgNP (**D**), and HDAgNP (**E**). All samples were stained directly on the substrates. Panel (**F**) shows the background normalized fluorescence intensity for each substrate type shown in the figure.

**Figure 4:**
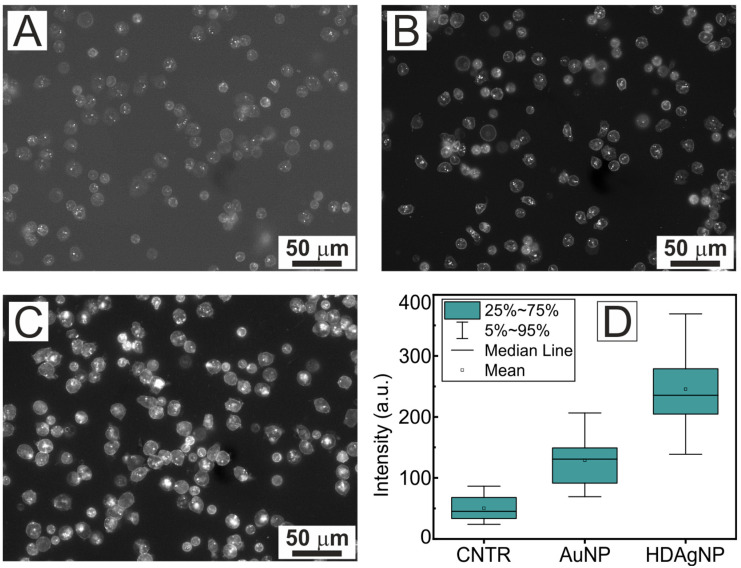
Fluorescence images of neutrophils seeded on different substrates: CNTR (**A**), AuNP (**B**), and HDAgNP (**C**). All samples were stained before placement on the substrates. Panel (**D**) shows the background normalized fluorescence intensity for all substrate types shown.

To evaluate the impact of different plasmonic substrates on the fluorescence response of stained neutrophil membranes, a wide-field fluorescence microscopy technique was employed. The averaged fluorescence intensity of the neutrophil membranes across the cell area was analyzed. To understand the mechanism of dye interaction with the substrate and cells during staining, two types of experiments were performed. In the first case, cells were stained directly on the plasmonic substrate (see Figure 3), and in the second, cells were stained in suspension immediately after isolation before seeding onto the plasmonic substrate (see Figure 4).

When cells were stained directly on the substrate, a comparison was made across five types of samples. The control sample (CNTR) consisted of borosilicate glass, a material commonly used for substrates. Additionally, a silver film deposited using PVD on a glass substrate (AgFilm) was measured, along with plasmonic substrates composed of layers of gold nanoparticles (AuNP) or silver nanoparticles (LDAgNP and HDAgNP) on glass substrates.

It has been demonstrated that when cells were stained directly on the substrates, the fluorescence intensity values were similar for CNTR, AgFilm, and AuNP samples, measuring at 50, 44, and 43 a.u., respectively (Figure 3F). However, on substrates with low density of silver nanoparticles (LDAgNP), the fluorescence response exhibited a significant enhancement of 3.5-fold (174 a.u.) compared to the control sample (CNTR). For the HDAgNP samples, the fluorescence enhancement of neutrophil membranes was even more pronounced, exceeding control values by more than 12-fold (638 a.u.). The observed enhancements for LDAgNP and HDAgNP substrates are comparable to or exceed previously reported fluorescence intensity enhancement [18,54].

It is important to note that all measured fluorescence intensity values were normalized to the background signal from residual unbound dye on the substrate. Additionally, only cells adhered to the substrate with non-spherical shapes and lower brightness (Figure 3A–E) were included in the analysis. Bright, round cells were excluded due to the distinct configurations of their membranes, which lead to higher brightness.

The results demonstrate that plasmonic substrates based on silver nanoparticles (LDAgNP and HDAgNP) significantly enhance the fluorescence response from membranes stained with WGA conjugated to Alexa Fluor 594. In contrast, the plasmonic substrate with gold nanoparticles, despite the close match between the plasmon absorption peak of the particles and the dye excitation band, did not demonstrate an enhancement of the fluorescence signal.

Furthermore, it was observed that neutrophils exhibited vesiculation if adhered to LDAgNP or HDAgNP substrates. Typically, vesiculation in neutrophils occurs following exposure to various stimulants, such as bacterial products, cytokines, chemokines, and exogenous compounds [55]. However, in this case, the vesiculation most probably occurred as a result of the mechanical stimulus from the substrates.

Additionally, a correlation was observed between the surface density of nanoparticles and the magnitude of the response in neutrophils (see Figure 3D,E). It is known that neutrophils exhibit responsiveness to variations in substrate material [30]. The observed effect of nanoparticle density is a potentially interesting and worthy of further investigation.

Figure 4 shows fluorescence images of cells (Figure 4A–C) and background-normalized fluorescence intensity values of neutrophils attached to the substrate (Figure 4D). The cells were stained before being seeded on the plasmonic substrates. It can be seen that for the gold AuNP sample, the fluorescence response of the neutrophils was much stronger when stained in suspension compared to cells stained directly on the substrate. Specifically, the background-normalized fluorescence intensity for the AuNP substrate was 2.5 times higher (128 a.u.) than that of the control glass sample (50 a.u.). Moreover, the high-density silver nanoparticle system (HDAgNP) exhibited a substantial fluorescence enhancement of 245 a.u., which is 4.9 times greater than the control sample and approximately twice that of the AuNP substrate.

The observed discrepancy between the two experimental setups can be attributed to the possibility that, during post-seeding staining, a portion of the fluorescent molecules may remain on the substrate surface despite thorough washing. This residual is likely to result in a higher concentration of fluorescent molecules in the space between the cell and the substrate surface compared to regions of the substrate not covered by cells.

An important characteristic of substrates with silver nanoparticles fabricated via the Na^+^-Ag^+^ ion exchange method is their capacity for reuse following mechanical surface cleaning. To evaluate the effectiveness of this process, the HDAgNP substrate, which demonstrated optimal performance during first use, was subjected to mechanical cleaning and reannealing (T = 500 °C for 3 h).

**Figure 5:**
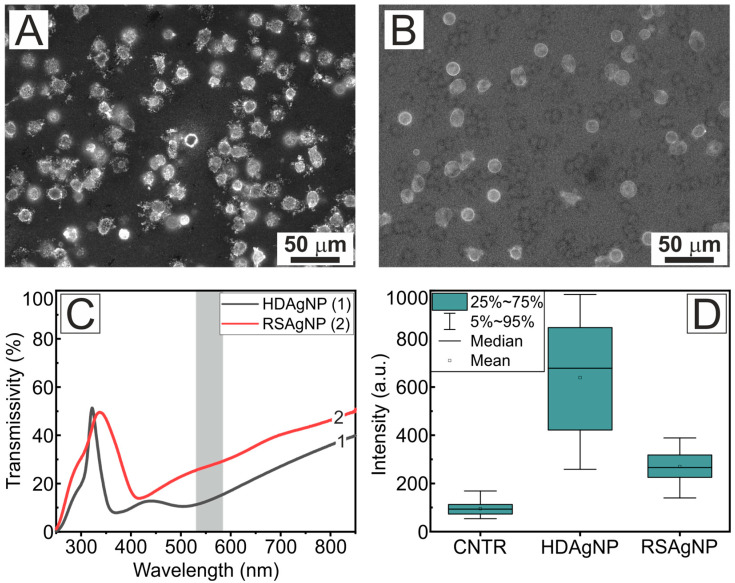
Fluorescence images of neutrophils on substrates with silver nanoparticles HDAgNP (**A**) and RSAgNP (**B**). All samples were stained directly on the substrates. Optical transmission spectra of HDAgNP (1) and RSAgNP (2) samples are shown in panel (**C**). Panel (**D**) shows the background normalized fluorescence intensity for the HDAgNP and RSAgNP samples.

Figure 5 shows fluorescent images of neutrophils on the HDAgNP substrate (Figure 5A) and the recovered RSAgNP substrate (Figure 5B). The transmission spectra of these substrates, along with the calculated average fluorescence intensity within the contour of adhered cells, are presented in panels C and D. One can see that after recovery, the transmission spectrum of the RSAgNP substrate exhibited other distinctive dips and higher transmittance values compared to the original HDAgNP substrate.

As can be seen in Figure 5D, the fluorescence intensity on the HDAgNP substrate was reduced by a factor of 4.2 after recovery, indicating a significant decrease in fluorescence response. Nevertheless, the recovered RSAgNP substrate exhibited a 1.5-fold increase in fluorescence intensity compared to the control glass sample. These findings suggest that it is feasible to reuse plasmonic systems based on silver nanoparticles prepared using the Na^+^-Ag^+^ ion exchange method after mechanical cleaning and reannealing. The glass substrate functions as a reservoir for silver ions, which form new silver nanoparticles during the recovery process. However, this leads to a substantial decline in fluorescence response, presumably due to a decrease in the plasmonic particle density. It is also important to note that the reannealing conditions were selected based on general considerations, and further investigation is necessary to optimize these parameters and maximize the fluorescence enhancement effect.

**Figure 6:**
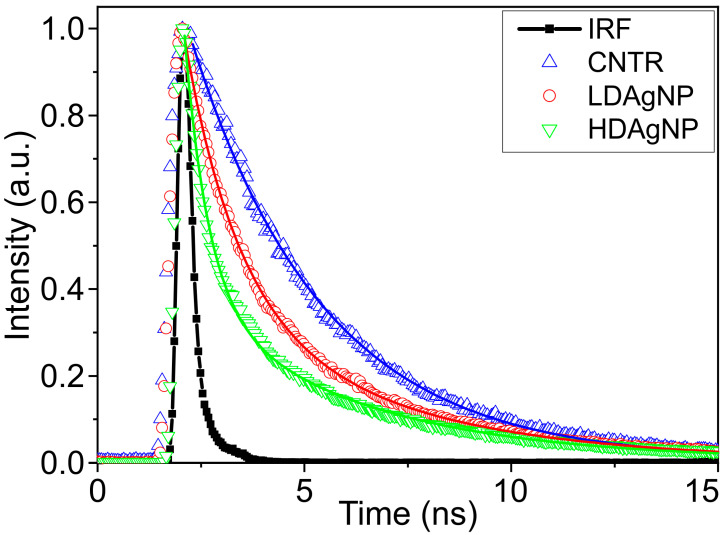
Fluorescence decay kinetics of WGA conjugated with Alexa Fluor 594 dye on substrates with silver nanoparticles (LDAgNP and HDAgNP) and on a glass substrate without nanoparticles (CNTRL). The instrument response function (IRF) is shown as a black line with filled squares for comparison.

To understand the mechanisms underlying fluorescence enhancement, it is essential to analyze the fluorescence decay kinetics of the dye in the vicinity of plasmonic nanostructures [21,56,57]. The presence of a plasmonic nanoparticle in close proximity to a fluorescent molecule can modify the rate constants of both radiative and non-radiative transitions within the molecule, thereby affecting the fluorescence intensity [58]. Furthermore, an increase in the effective oscillator strength of a fluorophore, related to an increase in the local density of optical states and energy transfer from plasmonic nanoparticles, may also impact fluorescence intensity and lifetime [59,60]. The decay kinetics of Alexa Fluor 594 dye conjugated to wheat germ agglutinin applied to low-density silver nanoparticle (LDAgNP) and high-density silver nanoparticle (HDAgNP) substrates and to a glass substrate without nanoparticles (CNTRL) are shown in Figure 6. On the glass substrate, the decay kinetics were characterized by a monoexponential function with a fluorescence lifetime of 3.17 ns. This measured lifetime is shorter than that of pure Alexa Fluor 594, which is 3.9 ns [61], due to conjugation with WGA.

The fluorescence decay kinetics measured on plasmonic substrates were found to be better approximated by biexponential functions. This complexity arises because the decay kinetics are influenced not only by the distance between the dye molecules and the surface of the silver nanoparticles but also by the individual sizes of the nanoparticles and their spatial distribution. Consequently, dye molecules on such substrates exhibit different fluorescence lifetimes.

The parameters of the decay curves are presented in Table 1. The average lifetime was calculated using the following equation to directly compare samples with multi-component decays:

$$\tau_{avg}=\frac{\sum_{i}A_{i}\tau_{i}^{2}}{\sum_{i}A_{i}\tau_{i}}$$

where $A_{i}$ is the amplitude and $\tau_{i}$ is the lifetime of component *i*.

The average lifetime of the dye on plasmonic substrates decreased by factors of 1.2 (for LDAgNP) and 2.8 (for HDAgNP). Furthermore, the lifetime of the short-lived component, which accounts for over 80% of the emission, decreased by factors of 2.5 and 5.5, respectively. These reductions in lifetime are well correlated with the observed increases in fluorescence intensity on substrates containing plasmonic nanoparticles.

## 4. Conclusions

It was demonstrated that the fluorescence intensity of neutrophil membranes stained with Alexa Fluor 594 conjugated with wheat germ agglutinin can undergo a more than 10-fold increase when the cells are located on glass substrates with large silver nanoparticles synthesized using the Na^+^-Ag^+^ ion exchange method. It is important to note that the degree of fluorescence enhancement is significantly affected by the size and density of the silver nanoparticles, as well as by the membrane staining protocol. In contrast, for substrates containing gold nanoparticles, a 2.5-fold enhancement in fluorescence intensity was only observed if neutrophils were stained prior to seeding onto the substrate. However, when staining was performed directly on the substrate, the fluorescence intensity was comparable to the control glass sample. This result can be explained by an increase in background fluorescence during on-substrate staining. Furthermore, a reduction in the fluorescence lifetime of the dye in the vicinity of plasmonic nanoparticles was observed. Specifically, the lifetime of the short-lived fluorescence component, responsible for over 80% of the emission, decreased by up to 5.5 times compared to the control sample.

The findings indicate that Na^+^-Ag^+^ ion-exchanged glass substrates are suitable for the real-time monitoring of cellular reactions while operating with lower concentrations of fluorescent dyes. The proposed method utilizes a Na^+^-Ag^+^ ion exchange process for fabricating plasmonic nanoparticles on a glass surface, allowing for the manipulation of the optical properties of the substrates in a wide range. The ion exchange process parameters, including the silver concentration in the salt melt, temperature, duration, external electric field, and subsequent substrate temperature treatment, can be adjusted to tailor the spectral properties for a specific fluorescent dye. It should be noted that in this study, the parameters were varied within narrow ranges; therefore, achieving further enhancement of fluorescence may require optimizing the ion exchange and annealing conditions.

## Figures and Tables

**Table 1:** Amplitude and lifetime estimated from exponential fit of Alexa Fluor 594 fluorescence decay kinetics (λex = 473 nm).

Sample	A_1_	τ_1_, ns	A_2_	τ_2_, ns	τ_AV_, ns
CNTR	1	3.171	-	-	-
LDAgNP	0.833	1.253	0.167	4.448	2.581
HDAgNP	0.974	0.579	0.026	4.098	1.138

## Data Availability

The datasets used and analyzed during the current study are available from the corresponding authors upon request.
